# Electronic Activation versus Steric Protection of a Rh(I) Pincer Complex inside a Supramolecular Metallobox

**DOI:** 10.1021/acs.inorgchem.6c02608

**Published:** 2026-07-13

**Authors:** Sebastián Martínez-Vivas, Susana Ibáñez, Eduardo Peris

**Affiliations:** Institute of Advanced Materials (INAM), Centro de Innovación en Química Avanzada (ORFEO−CINQA), Universitat Jaume I, Av. Vicente Sos Baynat s/n, 12006 Castellón, Spain

## Abstract

We report the formation of a highly stable host–guest assembly between a nanosized iridium­(I)-based metallobox and the square-planar pincer complex [Rh^I^(CNC)­(CO)]^+^ (with CNC being a planar pyridine-centered di-N-heterocyclic carbene pincer ligand). NMR and UV–vis studies reveal an exceptionally high binding affinity (*K*_a_ = 2.5 × 10^6^ M^–1^) in methylene chloride. Infrared spectroscopy shows that encapsulation within the electron-rich cavity increases the electron density at the rhodium center, as evidenced by a lowering of the CO stretching frequency. Despite this electronic enrichment, the confined Rh­(I) complex becomes remarkably resistant toward oxidative addition with MeI, demonstrating the strong protective effect exerted by the host cavity. These findings highlight how supramolecular confinement can simultaneously tune the electronic structure and reactivity of transition-metal complexes, offering new perspectives for the design of confined catalytic systems.

## Graphical abstract

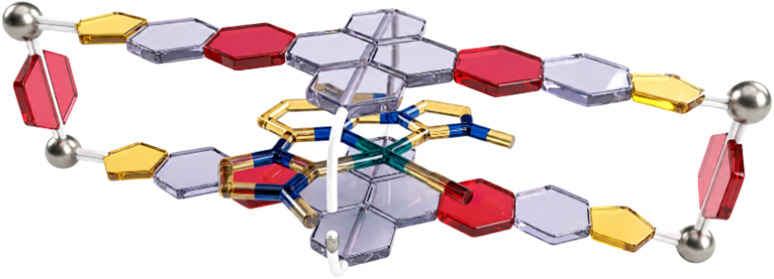

## Introduction

The ability to incorporate guest species within suitably designed molecular hosts provides a unique opportunity to investigate confinement effects arising from sterically and electronically restricted environments.^1^ Chemistry occurring within confined spaces results from a complex interplay of interactions that often extend beyond the individual molecule and cannot be readily attributed to a single factor. As a consequence, confined chemical systems frequently display reactivity patterns that differ significantly from those of their unconfined counterparts. In particular, the encapsulation of metal complexes within the cavities of artificial hosts can profoundly modify their reactivity^1a,1e,2^ and catalytic^3^ behavior, while also enabling the stabilization of otherwise highly reactive species.^4^

Over the past few years, we have focused on the development of a variety of organometallic metallasupramolecular assemblies capable of encapsulating diverse organic and organometallic guest molecules.^5^ Among the systems we have developed, our recently reported iridium-conjoined nanorectangle (**1**, Scheme 1) has proven particularly noteworthy because of its ability to encapsulate polycyclic aromatic hydrocarbons with exceptionally high binding affinities.^6^ In addition, this system provides an excellent platform for investigating the dynamics of the large-amplitude translational motions exhibited by guest molecules as they shuttle between the two sides of the nanoscale cavity.^6,7^ The study of such dynamic processes in host–guest systems is highly relevant to the bottom-up molecular design of molecular machines.^8^

**Scheme 1:**
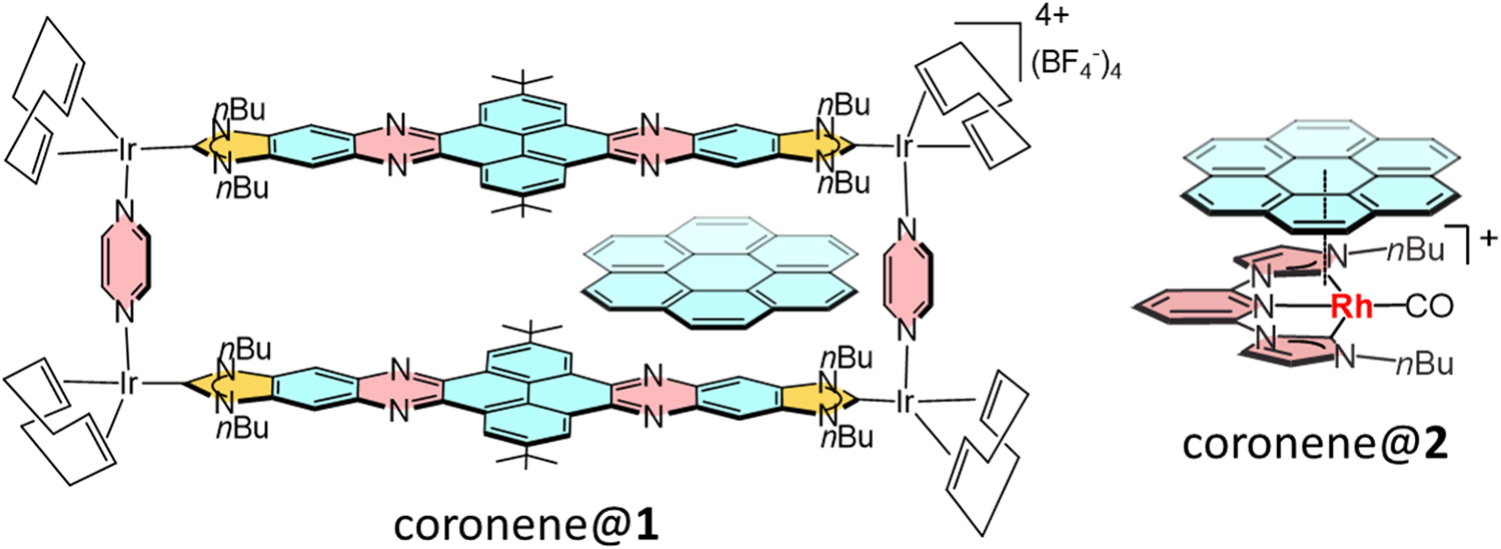
Structures of Metallobox **1** and the Rh­(I) Pincer Complex **2**, Illustrating Their Respective Interactions with Coronene

In parallel with these studies, we became interested in exploring how noncovalent π-stacking interactions can be exploited to modulate the reactivity and catalytic properties of square-planar metal complexes. In particular, we found that the cationic Rh­(I) complex [Rh­(CNC)­(CO)]^+^ (**2**, Scheme 1) displays a high binding affinity for coronene in CH_2_Cl_2_, and that this interaction exerts a pronounced influence on both the reactivity and catalytic behavior of the complex.^9^ These findings highlight the significant role that supramolecular interactions can play in tuning the reactivity and catalytic performance of square-planar metal complexes.

Building on these findings, we envisioned that combining the strong affinity of complex **2** for coronene with the high binding affinity exhibited by metallorectangle **1** toward the same polycyclic aromatic hydrocarbon could provide an effective strategy for promoting the association between **1** and **2**. By applying the transitive principle to molecular recognition, we reasoned that the mutual affinity of both systems for coronene should translate into a strong host–guest interaction between the metallorectangle **1** and complex **2**. Herein, we describe the remarkable binding affinity between metallobox **1** and the Rh­(I) complex **2**, and demonstrate how encapsulation within the confined cavity induces significant changes in the electronic structure and chemical properties of the metal complex relative to those of the unconfined species.

## Results and Discussion

The ^1^H NMR spectrum obtained from the addition of one equivalent of the pincer Rh­(I) complex **2** to a CD_2_Cl_2_ solution of metallobox **1**, revealed significant perturbations in the resonances corresponding to both **1** and **2**, strongly suggesting the effective encapsulation of **2** within the hollow cavity of the metallobox and therefore confirming the formation of **2**@**1**. In particular, addition of the guest molecule induced an upfield shift of the two resonances assigned to the aromatic protons of the horizontal polyaromatic panels (*a*′ and *b*′, relative to *a* and *b*, in Figure 1). In contrast, the resonance corresponding to the four equivalent protons of the pyrazine ligands experienced only a negligible shift, suggesting that the interaction between the guest molecule and the metallobox occurs mainly at the center of the cavity, far from the pyrazine ligands located at its edges. With respect to the guest molecule, the resonances associated with the pincer ligand of the Rh­(I) complex were also significantly shifted. In this case, however, the signals appeared considerably broadened as a consequence of the dynamic behavior of the guest within the cavity of the metallobox. Since the signals corresponding to the pyrene protons of the metallobox also became broadened, we propose that the dynamic process involves a rocking motion of the guest molecule within the host cavity. This behavior likely arises from the presence of two degenerate configurations that differ in the relative orientation of the guest inside the host cavity, as illustrated in Figure 2. When the interconversion between these conformations becomes slow on the NMR time scale, the 2-fold symmetry of the host is effectively broken, rendering the protons of the polyaromatic panels inequivalent.

**Figure 1:**
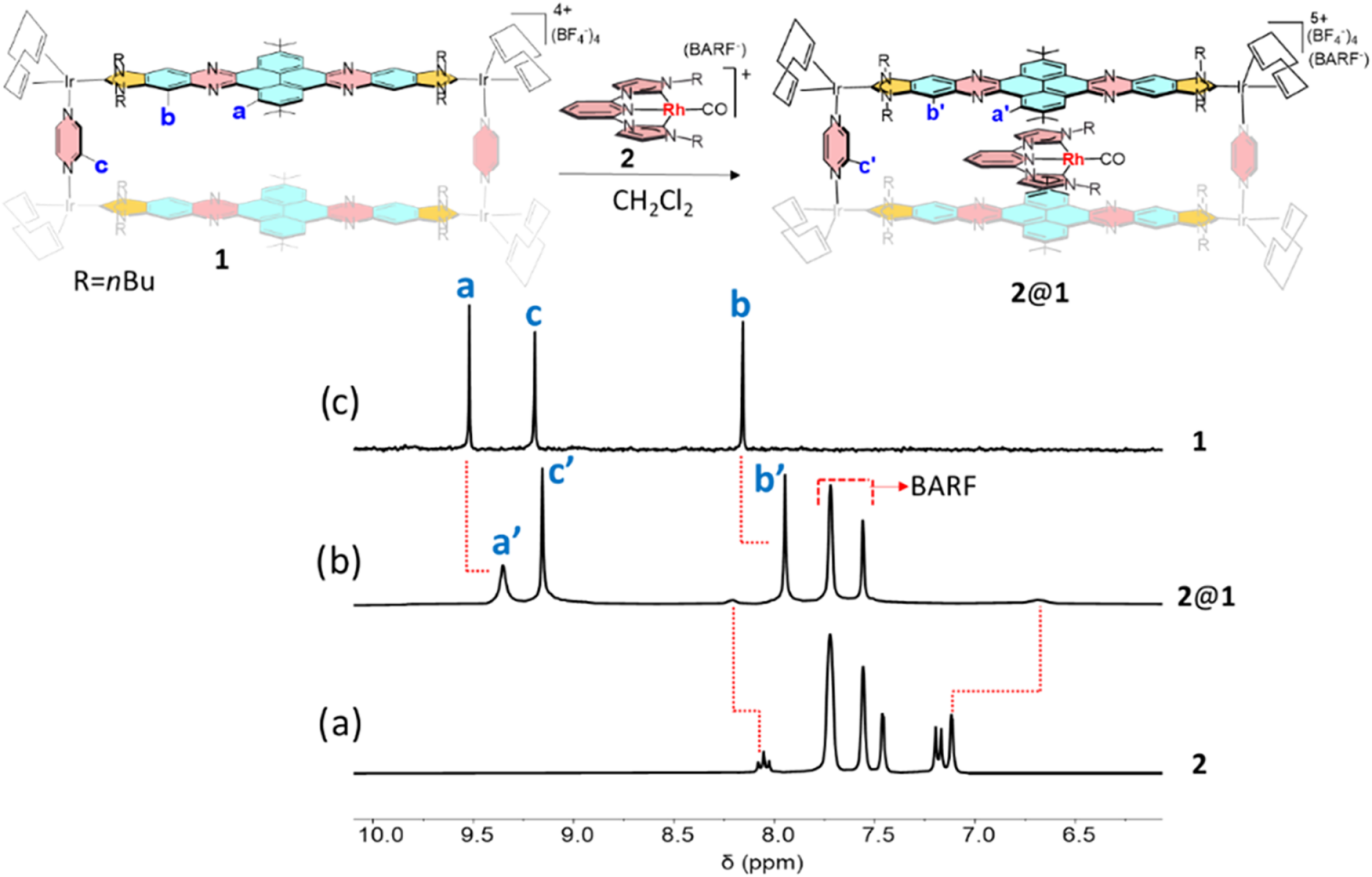
Selected region of the ^1^H NMR spectrum (5 mM, CD_2_Cl_2_, 500 MHz, 298 K) of (a) **2**, (b) **2**@**1**, and (c) **1**.

**Figure 2:**
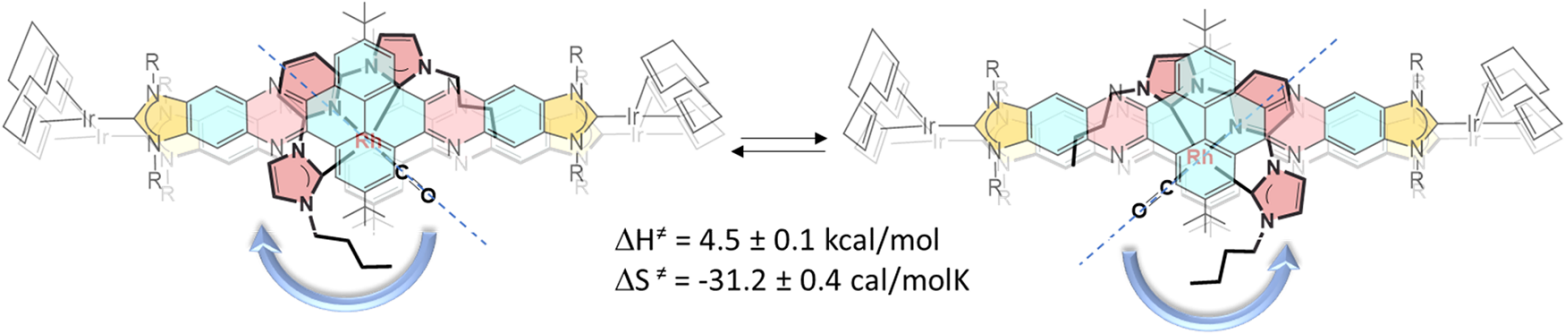
Schematic representation of the dynamic rocking motion undergone by the encapsulated Rh­(I) pincer complex within **2**@**1**.

To investigate this dynamic behavior, variable-temperature ^1^H NMR experiments were performed in CD_2_Cl_2_. As shown in the series of spectra presented in Figure S10 of the Supporting Information, the transition between the fast- and slow-exchange regimes occurs at 273 K for pyrene resonance *a*′, at 213 K for the pyrazine and benzene resonances (*c*′ and *b*′, respectively), and at 243 K for the resonance corresponding to the methyl groups of the *tert*-butyl substituents located on the sides of the pyrene units. Lineshape analysis of the spectra allowed extraction of the exchange rates at each temperature, using the signals of the *tert*-butyl methyl protons as reference. Subsequent Eyring analysis afforded the activation parameters: an enthalpy of activation (Δ*H* ^‡^) of 4.5 ± 0.1 kcal mol^–1^ and an entropy of activation (Δ*S* ^‡^) of −31.2 ± 0.4 cal mol^–1^ K^–1^, corresponding to a Gibbs free energy of activation (Δ*G* ^‡^) of 13.8 kcal mol^–1^ at 298 K. The positive enthalpy likely reflects the energetic cost associated with the structural rearrangement required for exchange, whereas the negative entropy indicates a more ordered transition state relative to the ground state, possibly due to increased host–guest organization or solvent structuring during the process.

For the determination of the binding affinity between **1** and **2**, ^1^H NMR titration experiments were unsuitable, because the very high binding affinity between the metallobox host and the Rh­(I) guest leads to essentially quantitative complex formation even at low concentrations and upon addition of substoichiometric amounts of guest, thereby preventing accurate determination of the association constant by NMR methods.

UV–vis titration experiments instead provided a binding constant of (2.5 ± 0.4) × 10^6^ M^–1^, which is the highest value we have observed for any guest encapsulated by metallobox **1**.^6,7^ This result clearly highlights the potential of this host system for the sequestration of pseudosquare-planar homogeneous catalysts. It is important to note that all titrations were performed at a constant concentration of **1** to minimize any influence of possible host self-association on the determination of the binding constant. To further confirm the exceptionally high affinity between **1** and **2**, a competitive binding experiment was performed in which one equivalent of pincer rhodium complex **2** was added to a CD_2_Cl_2_ solution of coronene@**1**. The resulting ^1^H NMR spectrum showed complete displacement of the coronene guest by **2** from the interior of the host cavity (see ESI for full details). This observation is particularly significant considering that coronene itself binds strongly to 1 in CD_2_Cl_2_, with an association constant of 1.8 × 10^5^.^6^ The superior affinity of 2 is likely a consequence of its excellent size and shape complementarity with the cavity of 1, which allows the Rh­(I) pincer complex to occupy the electron-rich central region of the host. In this arrangement, the cationic charge of the complex can be more effectively stabilized, leading to substantially stronger host–guest interactions than those observed for coronene. It should also be noted that ^1^H NMR spectra of both **1** and **2** were recorded over a range of concentrations to assess the possibility of self-association. In neither case were any concentration-dependent changes observed in the spectra, indicating that neither **1** nor **2** undergoes detectable self-association under the conditions employed. Consequently, the unusually high binding affinity cannot be attributed to aggregation phenomena involving either species.

Next, we sought to investigate whether formation of **2**@**1** has any effect on the electron richness of the Rh­(I) center in **2**. To address this question, we recorded the infrared spectrum of **2**@**1** in CH_2_Cl_2_ and compared it with that of the free complex **2**. As shown in Figure 3, encapsulation of **2** within metallobox **1** is accompanied by a decrease of 5 cm^–1^ in the C–O stretching frequency, indicating an increase in the electron density at the rhodium center upon host–guest complex formation. This observation is consistent with the metal complex being positioned at the center of the cavity, where it experiences the strongest electronic influence from the electron-rich pyrene panels of the host. In principle, the increased electron richness of the metal center should enhance its tendency to undergo oxidative addition reactions, as we previously demonstrated.^9^

**Figure 3:**
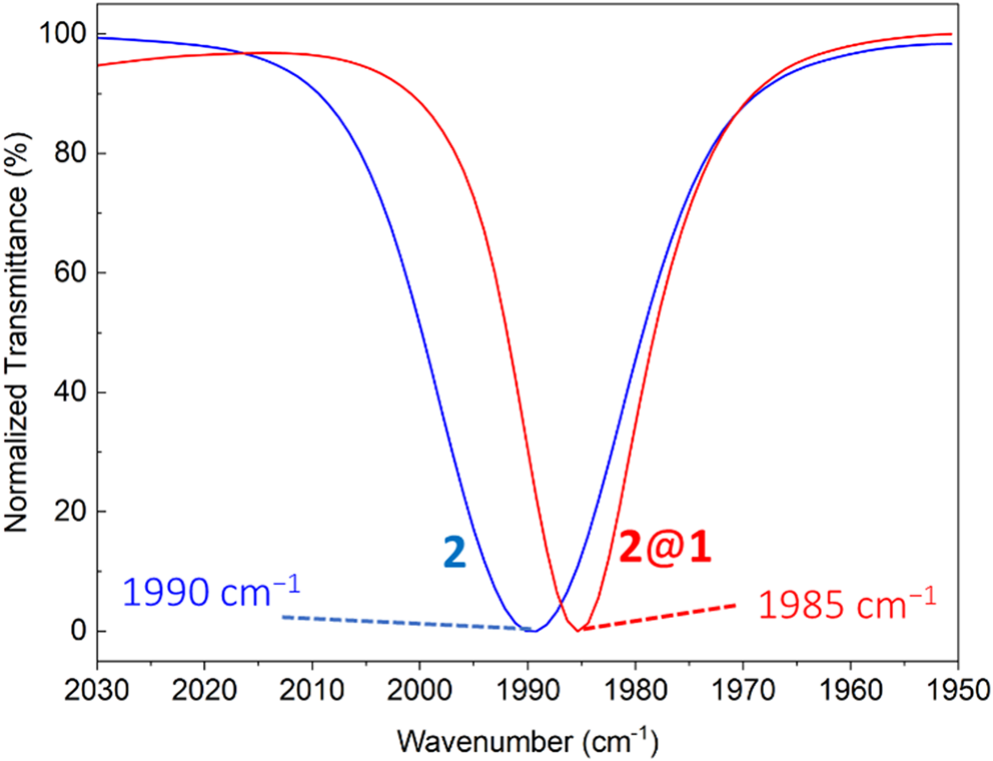
Infrared (IR) spectra of **2** (blue line), and **2**@**1 (red line)**, in CH_2_Cl_2_.

In order to investigate whether encapsulation of **2** affects its ability to undergo oxidative addition, we performed a comparative study of the reaction of **2** with MeI in the absence and presence of metallobox **1**. The reactions were carried out in CD_2_Cl_2_ at 40 °C using an excess of MeI relative to the rhodium complex (Rh/MeI = 1/48). Prior to these experiments, a control reaction was performed to determine whether the Ir­(I)-based metallobox **1** itself undergoes oxidative addition with MeI under the reaction conditions employed. No reaction was observed, confirming the stability of the metallobox.

As shown in Figure 4, the reaction between free complex **2** and MeI follows a pseudo-first-order kinetic profile and reaches completion after approximately 12 h. In contrast, when the reaction was carried out in the presence of one equivalent of metallobox **1**, no formation of the corresponding Rh­(III) complex was detected. Furthermore, when the reaction was performed using a Rh/**1** molar ratio of 2:1, the reaction rate was significantly reduced compared to that observed for free **2**, and only 50% conversion to the Rh­(III) product was achieved. This result is consistent with selective oxidation of only the nonencapsulated Rh­(I) complex, while the fraction confined within the cavity remains unreactive toward MeI.

**Figure 4:**
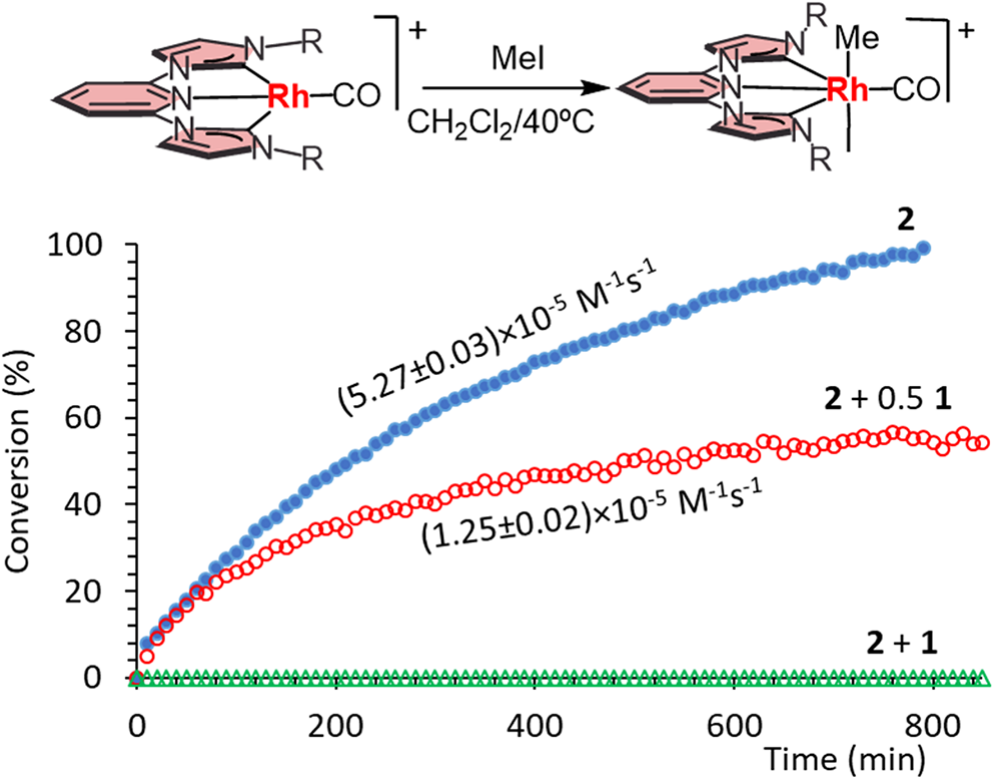
Time-dependent reaction profiles of the reactions of **2** with MeI in the presence and in the absence of metallobox **1**. The reactions were carried out in CD_2_Cl_2_ at 40 °C, using an initial concentration of the [Rh­(CNC)­(CO)]^+^ complex of 2 mM and a concentration of MeI of 96 mM. The reaction progress was monitored by ^1^H NMR. The blue solid dots represent the evolution of the reaction carried out with **2** alone, while the red empty circles represent the reaction in the presence of 0.5 equiv of **1**. The green, empty triangles correspond to the reaction performed with one equivalent of **1**, under which conditions no formation of Rh­(III) oxidative addition product was detected.

Overall, these results demonstrate that, despite providing a more electron-rich environment around the metal center, encapsulation of the pincer Rh­(I) complex within the cavity of **1** effectively protects the square-planar Rh­(I) species from oxidative addition by MeI.

## Conclusions

In summary, we have demonstrated that the iridium-based metallobox **1** displays an exceptionally high binding affinity toward the square-planar Rh­(I) pincer complex **2**, leading to the formation of a remarkably stable host–guest assembly. This large binding affinity highlights the strong complementarity between the electron-rich cavity of the host and the aromatic surface of the Rh­(I) complex. Encapsulation of the Rh­(I) complex within the cavity of **1** was also shown to significantly influence its electronic properties. In particular, the observed decrease in the CO stretching frequency demonstrates that confinement within the electron-rich pyrene-lined cavity increases the electron density at the rhodium center through noncovalent interactions. These results provide clear evidence that supramolecular encapsulation can effectively modulate the electronic structure of a transition-metal complex without altering its primary coordination sphere.

Despite the increased electron richness of the encapsulated metal center, confinement within the metallobox strongly suppresses its oxidative addition reactivity toward MeI. Whereas the free Rh­(I) complex is readily oxidized under the reaction conditions employed, the encapsulated complex remains completely unreactive. This protective effect highlights the ability of the host cavity to sterically shield the metal center from external reagents, thereby overriding the enhanced intrinsic reactivity expected from the more electron-rich environment.

Overall, this work demonstrates how supramolecular confinement can simultaneously modulate the electronic properties and chemical reactivity of a transition-metal complex through a subtle balance of steric and electronic effects. These findings open new opportunities for the design of confined catalytic systems in which molecular hosts can be used to control catalyst activity, selectivity, stability, and substrate accessibility through noncovalent interactions.

## Experimental Section

### General Considerations

The metallobox **1**, and [Rh­(CNC)­(CO)]­(BAr_4_^F^) (**2**) CNC = 2,6-bis­(1-*n*-butylimidazolium-3-yl)­pyridine were prepared according to literature methods. All other reagents were used as received from commercial suppliers. NMR spectra were recorded on a Bruker 300 MHz and Varian Innova 500 MHz using CD_2_Cl_2_ as solvents. VT-NMR spectra were recorded from 263 to 323 K on a Varian Innova 500 MHz. Elemental analyses were carried out on a TruSpec Micro Series. Infrared spectra (FTIR) were performed on a Bruker Equinox 55 FTIR spectrometer with a spectral window 4000–400 cm^–1^. BindFitv0.5 program was employed for the calculation of the association constants. UV-Vis absorption spectra were recorded on a Varian Cary 300 BIO spectrophotometer using dichloromethane under ambient conditions.

### Synthesis of **2**@**1**

A solution of the metallobox **1** (41.9 mg, 0.01 mmol) and [Rh­(CNC)­(CO)]­(BAr_4_^F^) (**2**) (16.45 mg, 0.01 mmol) in dichloromethane (20 mL) was stirred overnight at room temperature. The solution was concentrated to almost dryness. *N*-hexane was added to precipitate an orange solid. Yield: 44.9 mg (77%). Elemental analysis calcd (%) for C_200_H_217_N_25_OIr_4_RhB_5_F_40_.4CH_2_Cl_2_: C, 48.88; H, 4.52; N, 6.99. Found C, 48.32; H, 4.66; N, 7.03 ^1^H NMR (300 MHz, 298 K, CD_2_Cl_2_): δ 9.35 (s, 8H, C*H*_pyr_), 9.16 (s, 8H, C*H*_pyra_), 8.24–8.17 (m, 1H, C*H*_pyr‑Rh_), 7.95 (s, 8H, C*H*_quino_), 7.78–7.67 (m, 8H, Ar^F^ + 2H, C*H*_imid_), 7.60–7.52 (m, 4H, Ar^F^ + 2H, C*H*_pyr‑Rh_), 6.78–6.57 (m, 2H, C*H*_imid_), 5.55–5.44 (m, 8H, NC*H*_2_CH_2_CH_2_CH_3_), 4.95–4.81 (m, 4H, NC*H*_2_CH_2_CH_2_CH_3‑Rh_), 4.78–4.66 (m, 8H, NC*H*_2_CH_2_CH_2_CH_3_), 4.20 (br s, 8H, CH_COD_), 4.16 (br s, 8H, CH_COD_), 2.59–2.48 (m, 16H, NCH_2_C*H*_2_CH_2_CH_3_), 2.24–2.36 (m, 4H, NCH_2_C*H*_2_CH_2_CH_3‑Rh_), 2.24–2.10 (m, 16H, NCH_2_CH_2_C*H*_2_CH_3_+ 4H, CH_2_CH_2_C*H*_2_CH_3‑Rh_), 1.92–1.75 (m, 32H, C*H*_2COD_), 1.64 (s, 36H, C­(C*H*_3_)), 1.24 (t, ^3^J_H–H_ = 9 Hz, 24H, NCH_2_CH_2_CH_2_C*H*_3_), 0.99 (t, ^3^ *J*_H–H_ = 7.3 Hz, 6H, CH_2_CH_2_CH_2_C*H*_3‑Rh_). ^13^C NMR (75 MHz, 298 K, CD_2_Cl_2_): δ 193.79 (Ir-*C*_carbene_), 151.35 (*C*_pyr‑imid_), 149.36 (*C*H_pyra_), 146.56 (*C*H_pyr‑Rh_), 142.48 (*C*_q_), 138.29 (*C*_q_), 136.92 (*C*_q_), 135.22 (Ar^F^), 131.96 (Ar^F^), 129.49 (*C*_q_), 129.07 (*C*_q_), 126.81 (Ar^F^), 124.93 (*C*H_pyr_), 124.78 (*C*H_imid_), 123.20 (Ar^F^), 117.92 (Ar^F^), 117.80, (*C*H_imid_), 108.46 (*C*H_quino_), 106.78 (*C*H_pyr‑Rh_), 88.41 (*C*H_COD_), 68.99 (*C*H_COD_), 50.21 (N*C*H_2_CH_2_CH_2_CH_3_), 50.21 (N*C*H_2_CH_2_CH_2_CH_3‑Rh_), 36.12 (*C*(CH_3_)), 32.89 (NCH_2_ *C*H_2_CH_2_CH_3_), 31.72 (NCH_2_ *C*H_2_CH_2_CH_3‑Rh_), 32.40 (NCH_2_CH_2_ *C*H_2_CH_3_), 32.40 (NCH_2_CH_2_ *C*H_2_CH_3‑Rh_), 29.95 (C­(*C*H_3_)), 20.99 (*C*H_2COD_), 14.11 (NCH_2_CH_2_CH_2_ *C*H_3_), 14.11 (NCH_2_CH_2_CH_2_ *C*H_3‑Rh_). ^1^H NMR (500 MHz, 233 K, CD_2_Cl_2_): δ 9.47 (s, 8H, C*H*_pyr_), 9.13 (s, 8H, C*H*_pyra_), 8.35 (t, ^3^ *J*_H–H_ = 7.5 Hz, 1H, C*H*_pyr‑Rh_), 7.90 (s, 8H, C*H*_quino_), 7.76–7.65 (m, 8H, Ar^F^ + 2H, C*H*_imid_), 7.55 (br, 4H, Ar^F^), 7.48–7.34 (m, 1H, C*H*_pyr‑Rh_), 7.24–7.01 (m, 1H, C*H*_pyr‑Rh_), 6.83–6.68 (m, 2H, C*H*_imid_), 5.57–5.39 (m, 8H, NC*H*_2_CH_2_CH_2_CH_3_), 4.81–4.66 (m, 8H, NC*H*_2_CH_2_CH_2_CH_3_), 4.51–4.39 (m, 4H, NC*H*_2_CH_2_CH_2_CH_3‑Rh_), 4.21 (br s, 8H, CH_COD_), 4.08 (d, 8H, CH_COD_), 2.54–2.34 (m, 16H, NCH_2_C*H*_2_CH_2_CH_3_+ 4H, NCH_2_C*H*_2_CH_2_CH_3‑Rh_), 2.19–2.06 (m, 16H, NCH_2_CH_2_C*H*_2_CH_3_+ 4H, NCH_2_CH_2_C*H*_2_CH_3‑Rh_), 1.86–1.68 (m, 32H, C*H*_2COD_), 1.58 (d, 36H, C­(C*H*_3_)), 1.18 (m, 24H, NCH_2_CH_2_CH_2_C*H*_3_), 0.87 (m, 6H, CH_2_CH_2_CH_2_C*H*_3‑Rh_).

### ^1^H NMR Titration Experiments

^1^H NMR titration experiments were performed by adding increasing amounts of **2** (guest) to a solution of complex **1**. The experiment was carried out in CD_2_Cl_2_, at constant concentrations of the host (0.37 mM). Two solutions were prepared: solution A (only containing host at 0.37 mM) and solution B (containing host at 0.37 mM and guest at different mM). The addition of increasing amounts of solution B to solution A produced perturbations on the signal due to the proton of the pyrene, quinoxaline, or pyrazine core of the host. The association constants were determined by nonlinear least-squares analysis, by using the BindFitv0.5 program.^12^

### UV–Visible Titrations

UV–visible titration experiments were performed by adding increasing amounts of **2** (guest) to a solution of complex **1**. The experiments were carried out in degassed CH_2_Cl_2_, at constant concentrations of the host (1 × 10^–6^ M). Two solutions were prepared: solution A (only containing host) and solution B (containing host and guest at 1 × 10^–4^ M). The addition of increasing amounts of solution B to solution A produced a perturbation of the absorption spectra of the host. The association constants were determined by nonlinear least-squares analysis, by using the BindFitv0.5 program.^12^

## Supplementary Material



## Notes

Experimental details concerning the synthesis, characterization, and photophysical properties of **2**@**1**; titration experiments, data used for nonlinear fittings, VT-NMR studies, and all necessary NMR and UV–vis spectra; experiments related to the oxidative addition of **2** and **2**@**1** with MeI; and ^1^H NMR studies and reaction profiles (PDF)

## References

[1] Grommet A. B., Feller M., Klajn R. (2020). Chemical reactivity
under nanoconfinement. Nat. Nanotechnol..

[2] a Forgan, R. S. ; Lloyd, G. O. Reactivity in Confined Spaces 2021; Vol. 31, pp 1–469.

[3] Zhao L., Jing X., Li X. Z., Guo X. Y., Zeng L., He C., Duan C. Y. (2019). Catalytic properties
of chemical transformation within the confined pockets of Werner-type
capsules. Coord. Chem. Rev..

[4] Galan A., Ballester P. (2016). Stabilization of reactive species
by supramolecular encapsulation. Chem. Soc.
Rev..

[5] Ibáñez S., Poyatos M., Peris E. (2020). N-Heterocyclic carbenes: a door open
to supramolecular organometallic chemistry. Acc. Chem. Res..

[6] Ibáñez S., Salvà P., Dawe L. N., Peris E. (2024). Guest-shuttling
in
a Nanosized Metallobox. Angew. Chem., Int. Ed..

[7] Ibáñez S., Alemany-Chavarria M., Dawe L. N., Ujaque G., Peris E. (2025). Rocking (and
Shuttling) in a Nanosized Metallobox. Angew.
Chem., Int. Ed..

[8] Balzani V., Credi A., Raymo F. M., Stoddart J. F. (2000). Artificial
molecular machines. Angew. Chem., Int. Ed..

[9] Martínez-Vivas S., Poyatos M., Peris E. (2023). Supramolecular Control of the Oxidative
Addition as a Way To Improve the Catalytic Efficiency of Pincer-Rhodium
(I) Complexes. Angew. Chem., Int. Ed..

[12] Thordarson P. (2011). Determining
association constants from titration experiments in supramolecular
chemistry. Chem. Soc. Rev..

