# Supplementary material for: Electronic Activation versus Steric Protection of a Rh(I) Pincer Complex inside a Supramolecular Metallobox
Source: Inorg Chem. 2026 Jul 13;65(29):17215–20. doi: 10.1021/acs.inorgchem.6c02608 (PMC13418169; doi:10.1021/acs.inorgchem.6c02608)
Supplement: Supplementary file 1 [file ic6c02608_si_001.pdf]

Supporting information for:

Electronic Activation versus Steric Protection of a Rh(I) Pincer Complex inside a Supramolecular Metallobox

Sebastián Martínez-Vivas,[a] Susana Ibáñez,[a] and Eduardo Peris[a]*

[a]Institute of Advanced Materials (INAM). Centro de Innovación en Química Avanzada (ORFEO-CINQA). Universitat Jaume I. Av. Vicente Sos Baynat s/n. Castellón. E-12071. Spain. E-mail: eperis@uj.es

**1. Spectroscopic data**	**S2-S4**
1.1. ^{1}H, ^{13}C and ^{1}H-^{13}C HSQC NMR spectra of **2@1** in CD_2Cl_2	S2
**2. Infrared (IR) spectroscopy studies**	**S5**
**3. Titration experiments**	**S6-S9**
3.1.^{1}H NMR titration experiments	S6
3.2 UV-visible titrations	S8
**4. Competitive experiments**	**S10**
**5. Variable-temperature ^{1}H NMR experiments**	**S11-S12**
**6. DOSY experiment**	**S13-S14**
**7. Kinetic studies: oxidative addition of MeI**	**S14-S16**
**8. References**	**S16**

1. Spectroscopic data

1.1. ^{1}H, ^{13}C and ^{1}H-^{13}C HSQC NMR spectra of 2@1 in CD_2Cl_2

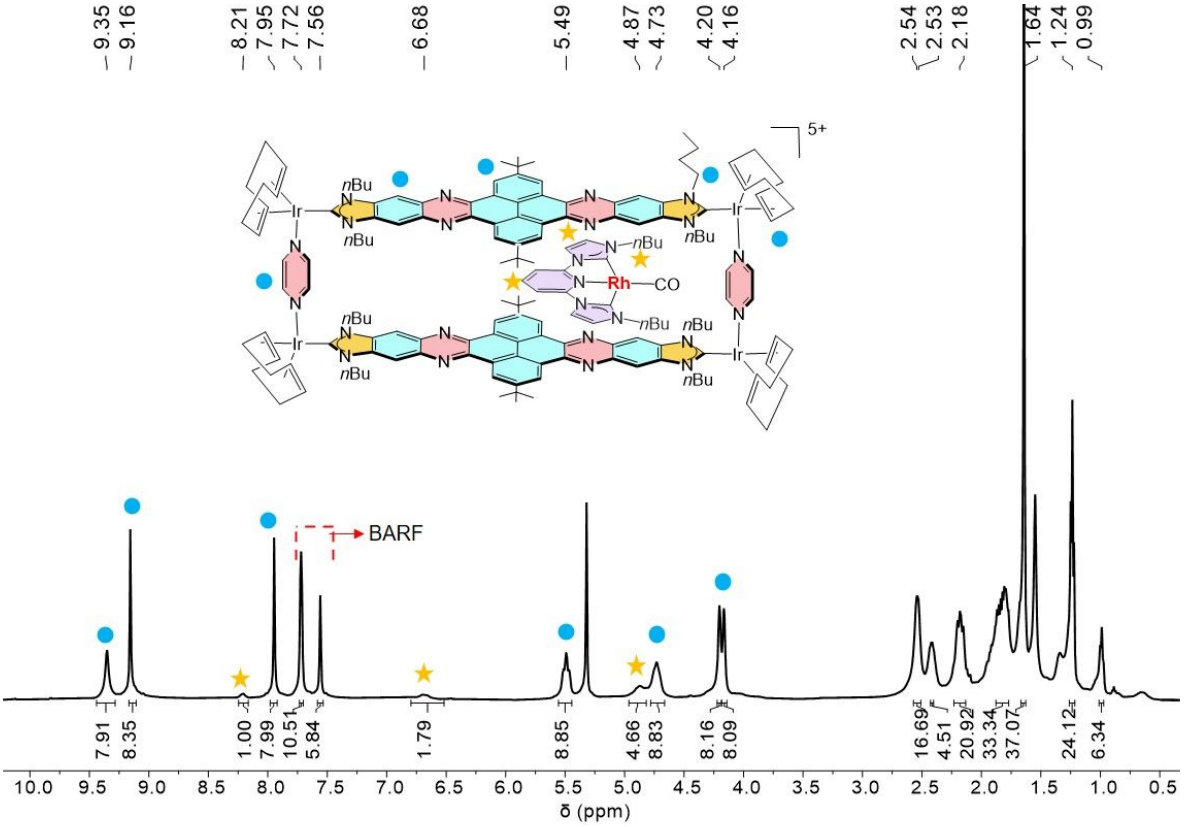

Figure S1. ^{1}H NMR spectrum (300 MHz) of **2@1** in CD_2Cl_2 at 298 K. Blue circles and yellow stars indicate the signals corresponding to the metallobox and to the encapsulated Rh(I) pincer complex, respectively.

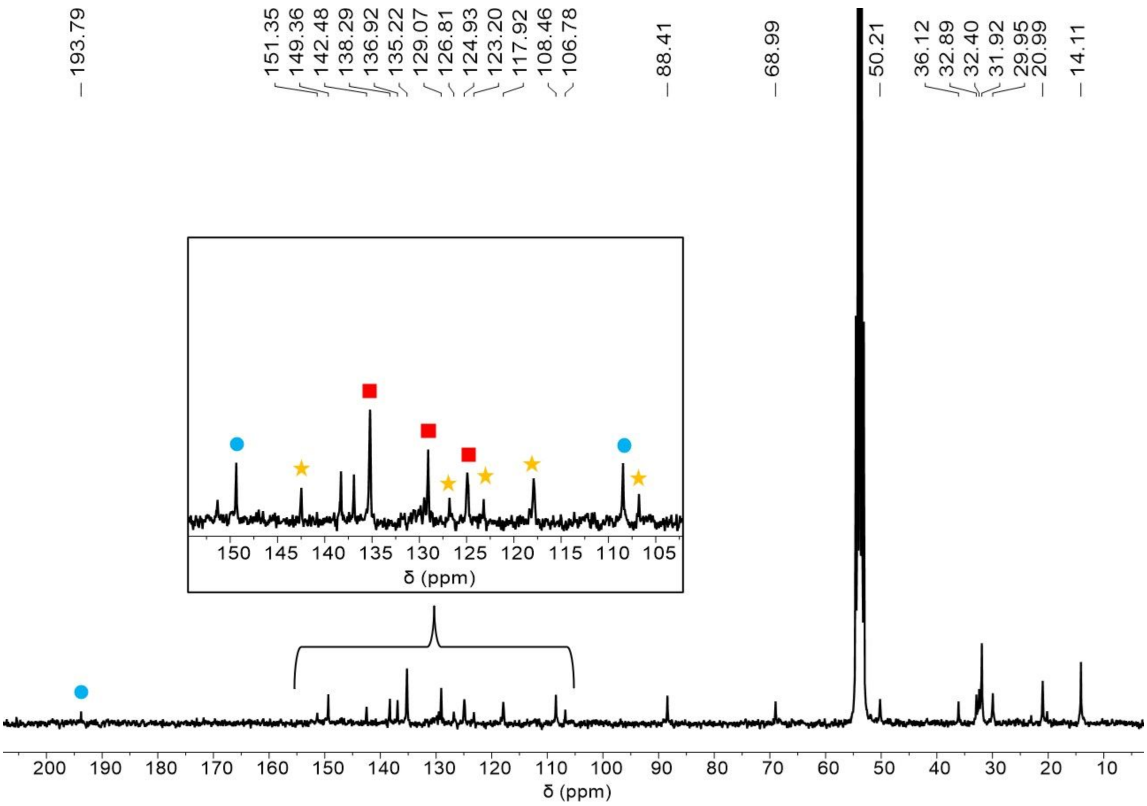

Figure S2. ^{13}C NMR spectrum (75 MHz) of **2@1** in CD_2Cl_2 at 298 K. Blue circles and yellow stars indicate the signals corresponding to the metallobox and to the encapsulated Rh(I) pincer complex, respectively. Red rectangles indicate the signals corresponding to the BARF anion.

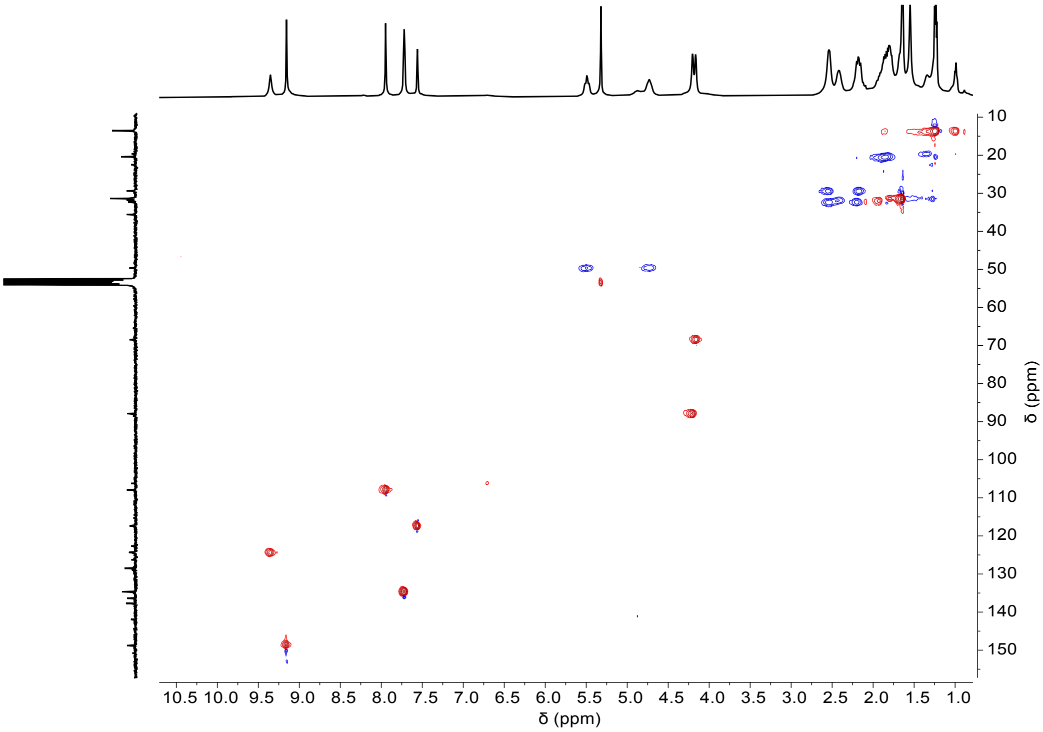

Figure S3. ^{1}H-^{13}C HSQC NMR spectrum (300 MHz) of **2@1** in CD_2Cl_2 at 298 K.

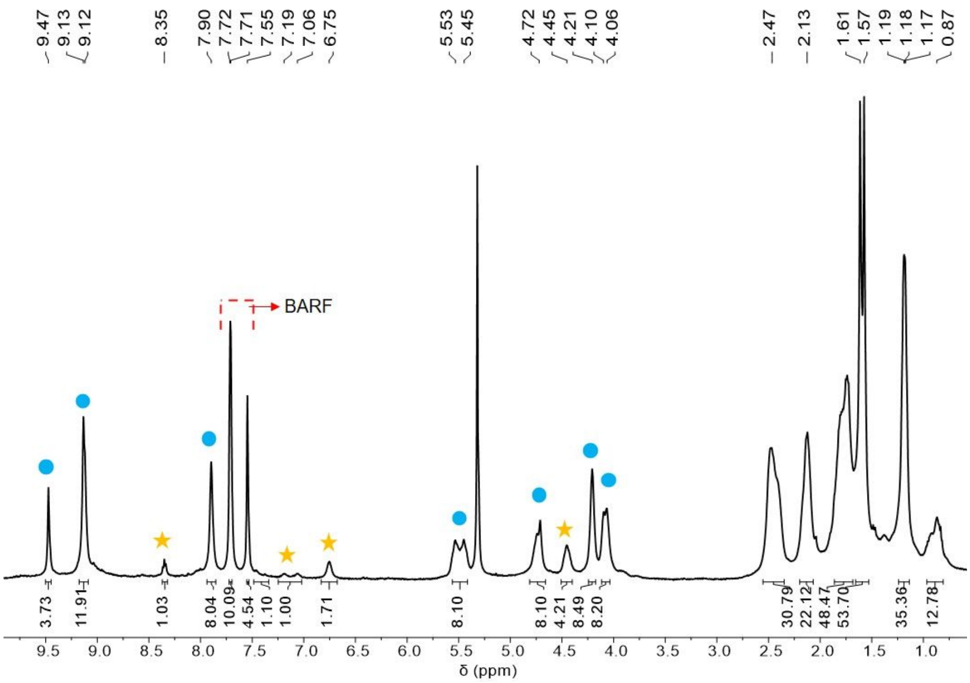

Figure S4. ^{1}H NMR spectrum (500 MHz) of **2@1** in CD_2Cl_2 at 233 K. Blue circles and yellow stars indicate the signals corresponding to the metallobox and to the encapsulated Rh(I) pincer complex, respectively.

2. Infrared (IR) spectroscopy studies

Samples of complex **2** and the host–guest assembly **2@1** were prepared in dry dichloromethane at a constant concentration of 2.5 mM. Encapsulation of **1** within **2** resulted in a shift of the C–O stretching frequency to lower wavenumbers, consistent with an increase in electron density at the metal center upon formation of the host–guest complex.

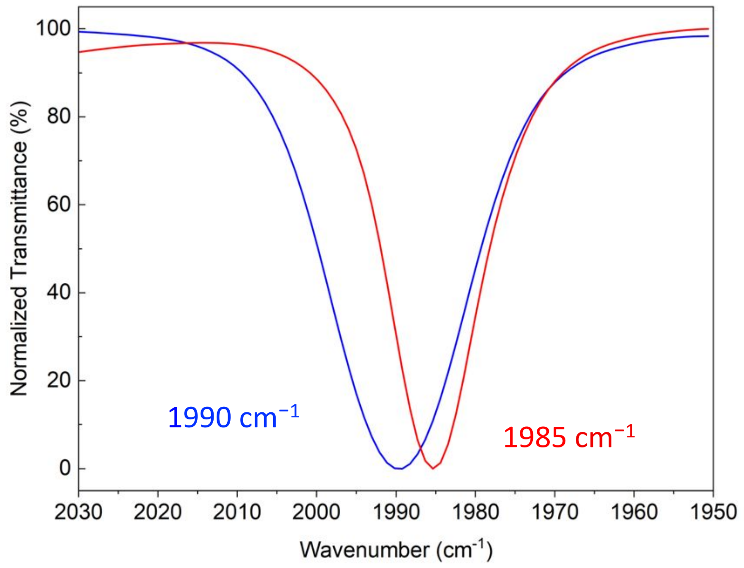

Figure S5. Selected region of the infrared spectra recorded for **2** (blue line) and **2@1** (red line) in CH_2Cl_2.

3. Titration experiments

3.1. ^{1}H NMR titration experiments

The recognition capability of metallobox **1** (host) was studied by ^{1}H NMR titration experiments, by adding increasing amounts of **2** (guest) to a solution of complex **1**. The experiment was carried out on CD_2Cl_2, at constant concentrations of the host (0.37 mM). Two solutions were prepared: solution A (only containing host at 0.37 mM) and solution B (containing host at 0.37 mM and guest at different mM). The addition of increasing amounts of solution B to solution A produced perturbations on the signal due to the proton of the pyrene, quinoxaline, or pyrazine core of the host. The association constants were determined by nonlinear least-square analysis, by using the BindFitv0.5 program.[1]

Titration of 1 with 2

Table S2. Data values from the titration study of **1** with **2**

[**1**] M	[**2**] M	δc_{Hpyr}	δc_{Hpyra}	δc_{Hquino}	equiv. **2**
0.00037255	0	9.52	9.18	8.16	0
0.00037255	6.5481E-05	9.51	9.18	8.15	0.2
0.00037255	0.00012844	9.45	9.17	8.05	0.4
0.00037255	0.00018903	9.39	9.17	8.01	0.5
0.00037255	0.00024737	9.37	9.17	7.97	0.8
0.00037255	0.0003578	9.36	9.16	7.96	1.0
0.00037255	0.00046062	9.36	9.16	7.96	1.2
0.00037255	0.00064636	9.36	9.16	7.95	1.7
0.00037255	0.00088399	9.36	9.15	7.95	2.4
0.00037255	0.00114246	9.35	9.15	7.95	3.0
0.00037255	0.00139793	9.35	9.14	7.95	3.7
0.00037255	0.00166975	9.35	9.14	7.95	4.5

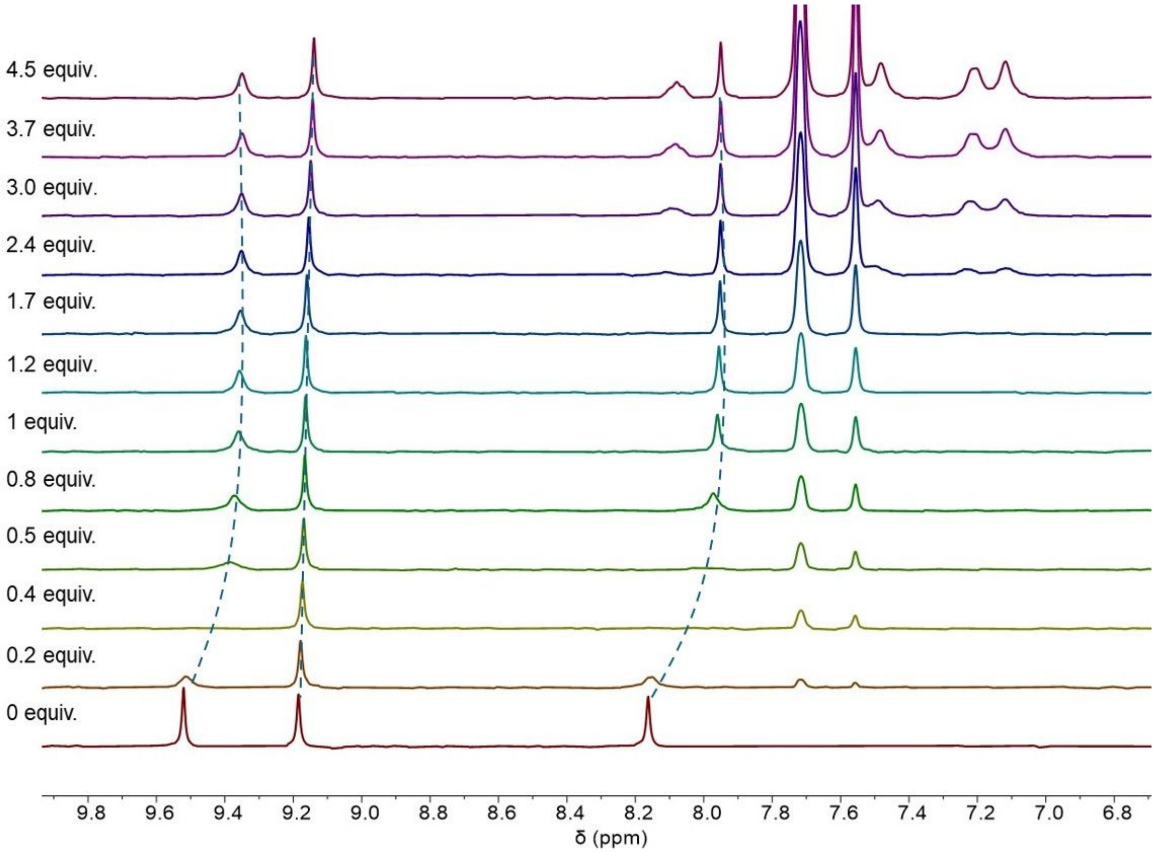

Figure S6. Selected region and spectra (CD_2Cl_2, 400 MHz, 298 K) of the titration of metallobox **1** (0.37 mM) with **2**. The line tracks the evolution of the chemical shift of the protons of **1** upon addition of increasing amounts of **2**.

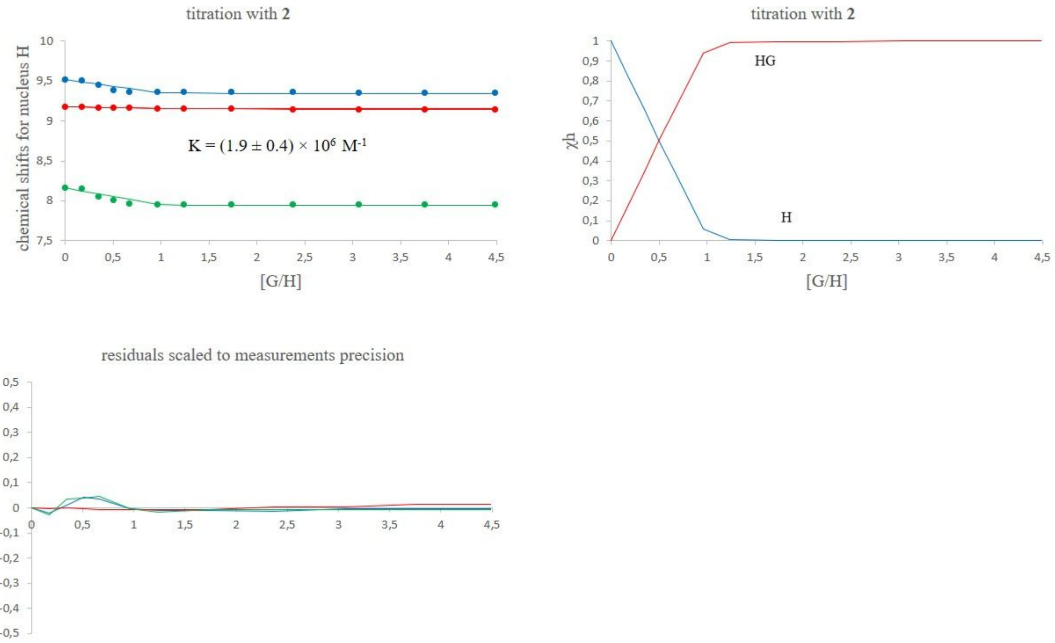

Figure S7. Non-linear least-squares fitting of the chemical shift changes of H during titration experiments of **1** with **2**. The Figure on the left represents the speciation profiles.

3.2. UV-visible titrations

The recognition capability of metallobox **1** (host) was studied by UV-visible titration experiments, by adding increasing amounts of **2** (guest) to a solution of complex **1**. The experiments were carried out in degassed CH_2Cl_2, at constant concentrations of the host ($1x10^{-6}$ M). Two solutions were prepared: solution A (only containing host) and solution B (containing host and guest at $1x10^{-4}$ M). The addition of increasing amounts of solution B to solution A produced a perturbation of the absorption spectra of the host. The association constants were determined by nonlinear least-square analysis, by using the BindFitv0.5 program.[1]

Titration of 1 with 2

Table S3. Data values from the titration study of **1** (H) with **2** (G)

[H] M	[G] M	[G]/[H]	A_{455nm}
1,0431E-06	0	0	0,13639
1,0431E-06	3,3032E-07	0,31665762	0,153
1,0431E-06	6,5845E-07	0,63121817	0,17998
1,0431E-06	9,8442E-07	0,94370242	0,20136
1,0431E-06	1,3082E-06	1,25413084	0,21997
1,0431E-06	1,6299E-06	1,56252367	0,23452
1,0431E-06	1,9495E-06	1,86890086	0,24179
1,0431E-06	2,267E-06	2,17328211	0,23989
1,0431E-06	2,5825E-06	2,47568686	0,23861
1,0431E-06	2,8959E-06	2,77613429	0,2463
1,0431E-06	3,2073E-06	3,07464335	0,24848
1,0431E-06	3,8241E-06	3,66592092	0,25053
1,0431E-06	4,433E-06	4,24966629	0,25669
1,0431E-06	5,0342E-06	4,82602248	0,25132
1,0431E-06	6,2141E-06	5,9571215	0,25833
1,0431E-06	7,3649E-06	7,06029214	0,25907
1,0431E-06	8,4876E-06	8,13655619	0,25509
1,0431E-06	9,5832E-06	9,18688616	0,26256
1,0431E-06	1,0653E-05	10,2122083	0,2628
1,0431E-06	1,1697E-05	11,2134052	0,26926
1,0431E-06	1,4204E-05	13,6162777	0,264
1,0431E-06	1,6571E-05	15,8856573	0,26328

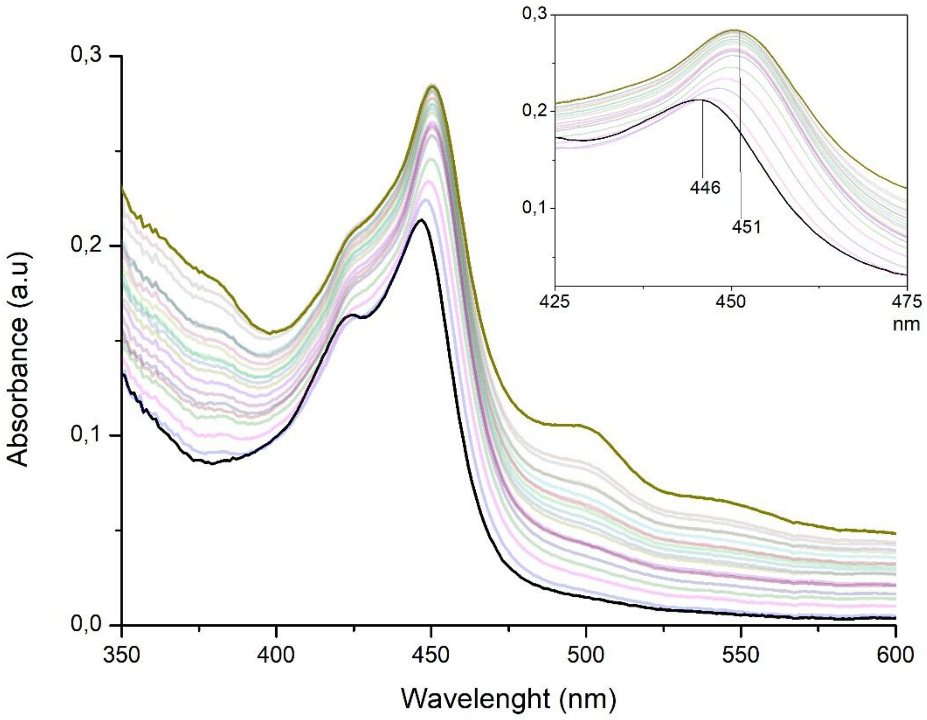

Figure S8. UV-visible absorption spectra acquired during the titration of **1** ($1x10^{-6}$ M) with **2** in CH_2Cl_2 at 298 K.

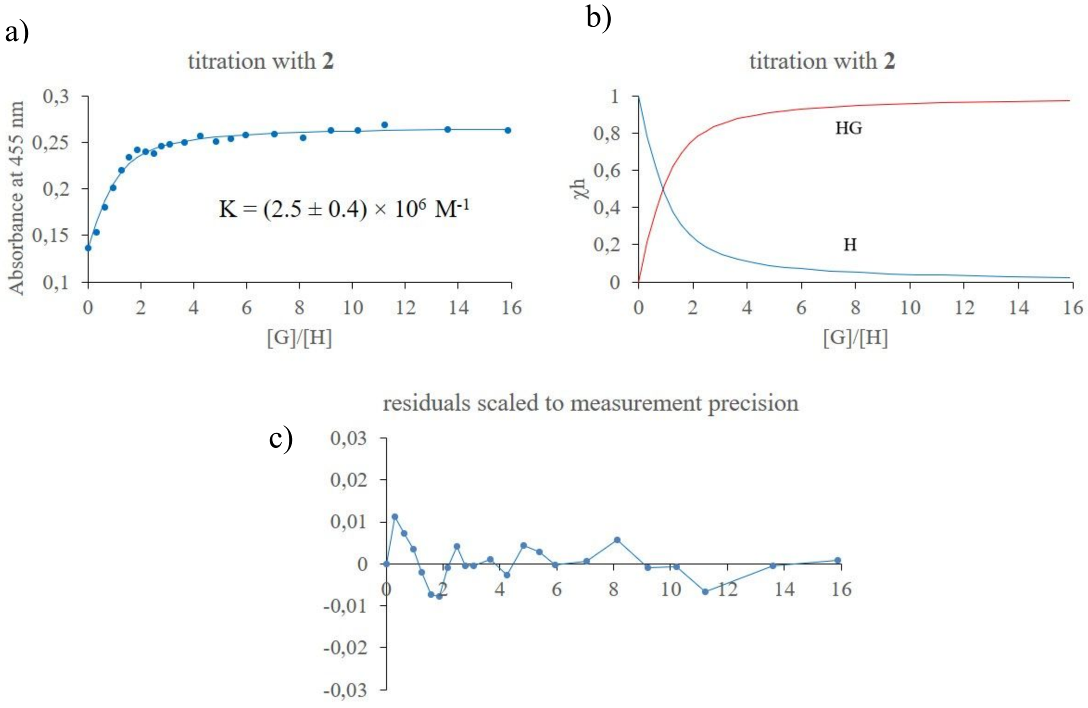

Figure S9. a) Non-linear least-squares fitting of the intensity absorption changes during titration experiments of **1** with **2**. b) The Figure on the left represents the speciation profiles. c) Residuals scaled to a measurement precision

4. Competitive experiments

An NMR tube was charged with 0.5 mL of a CD_2Cl_2 solution of coronene@**1** (1 mM). Then, 25 μL of a **2** (10 mM, 0.5 equiv.) were added. As can be seen in Figure S10b, upon the addition of 0.5 equivalent of **2** resulted in the formation of a metallobox **2@1,** coronene@**1** and free coronene. Subsequent addition of 25 μL of a **2** (10 mM, 1 equiv.) resulted in the formation of a metallobox **2@1**, and free coronene. (Figure S10c).

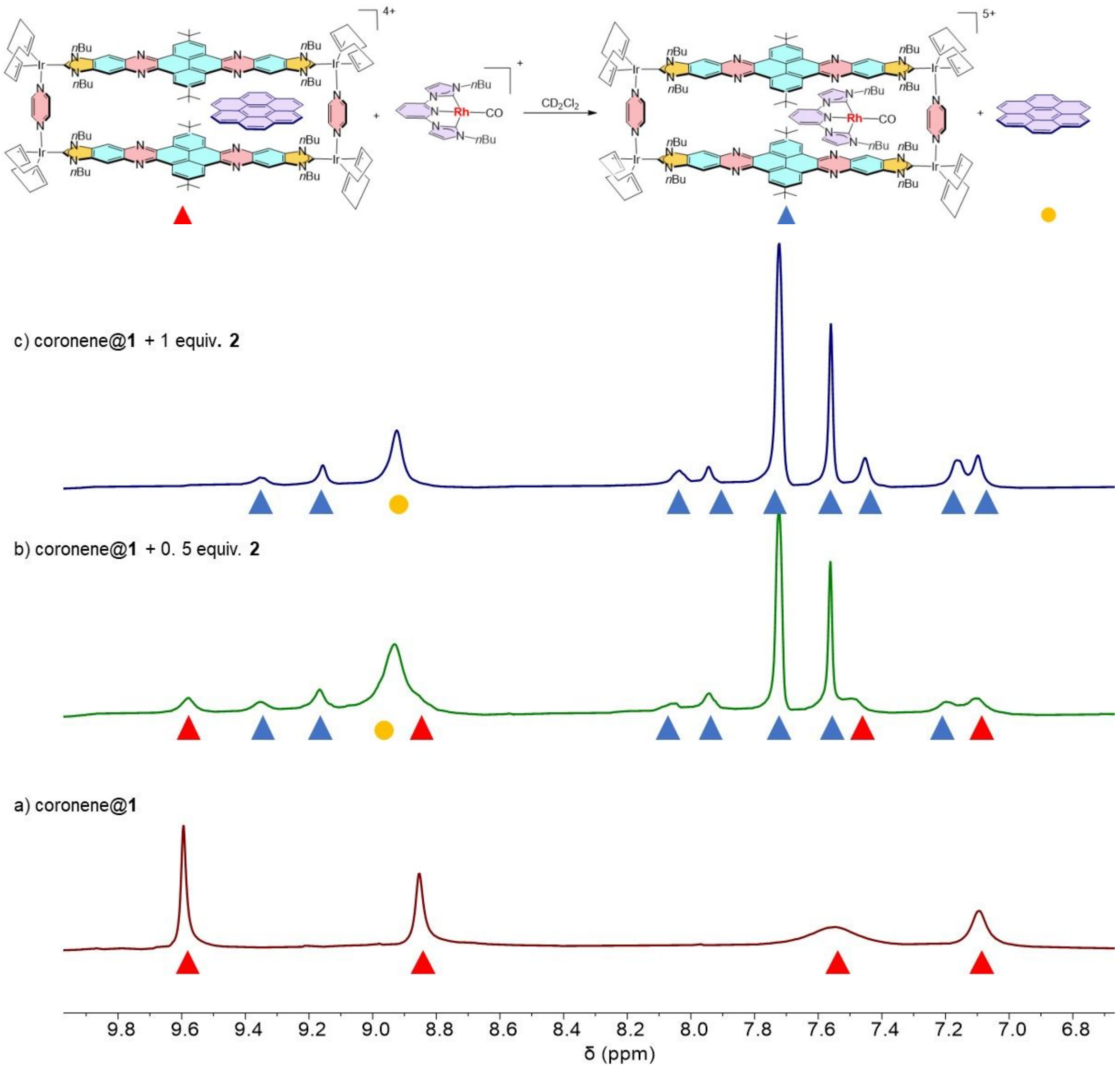

Figure S10. Selected region of the ^{1}H NMR spectra (CD_2Cl_2, 400 MHz, 298 K) of a) coronene@**1,** b) a coronene@**1** after the addition of 0.5 equiv. of **2**, and c) a coronene@**1** after the addition of 1 equiv. of **2**.

5. Variable-temperature ^{1}H NMR experiments

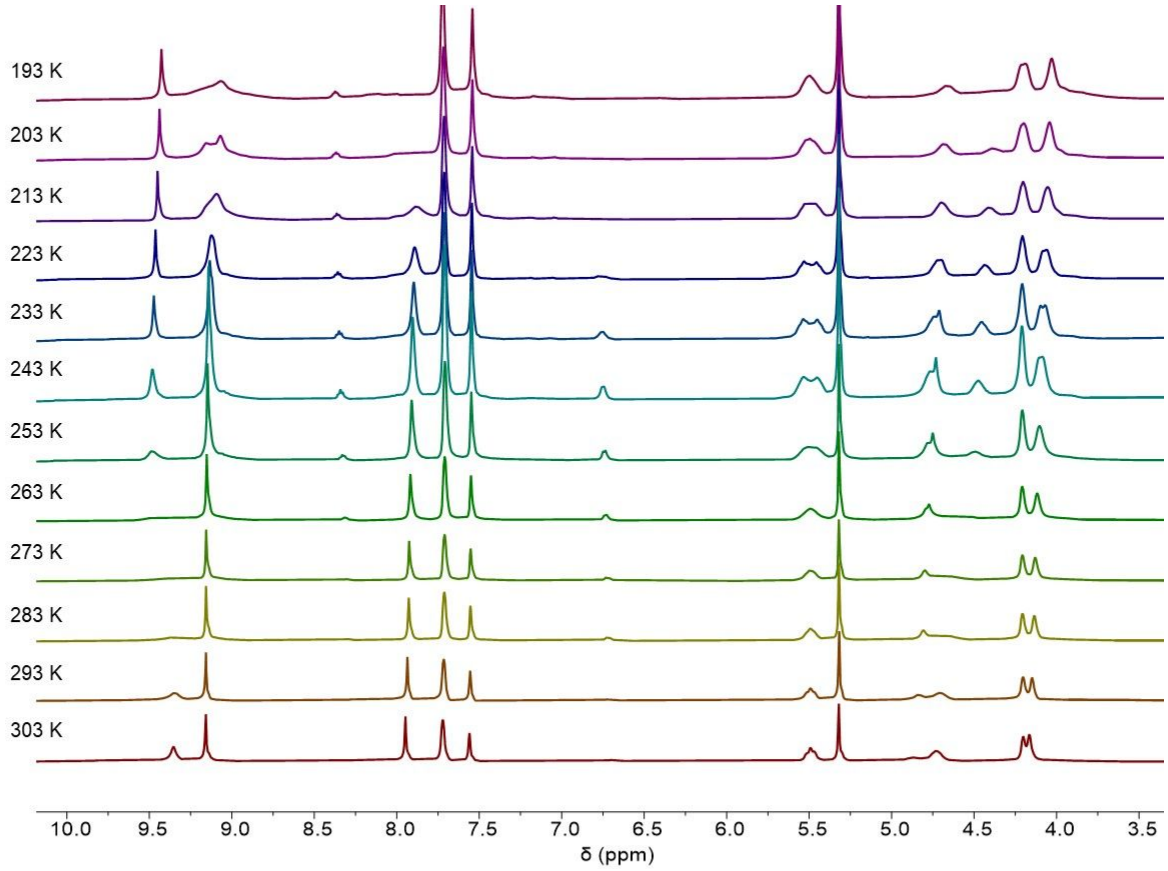

Figure S11. VT-^{1}H NMR studies (500 MHz) of complex **2@1** in CD_2Cl_2 with temperatures ranging from 193-303 K.

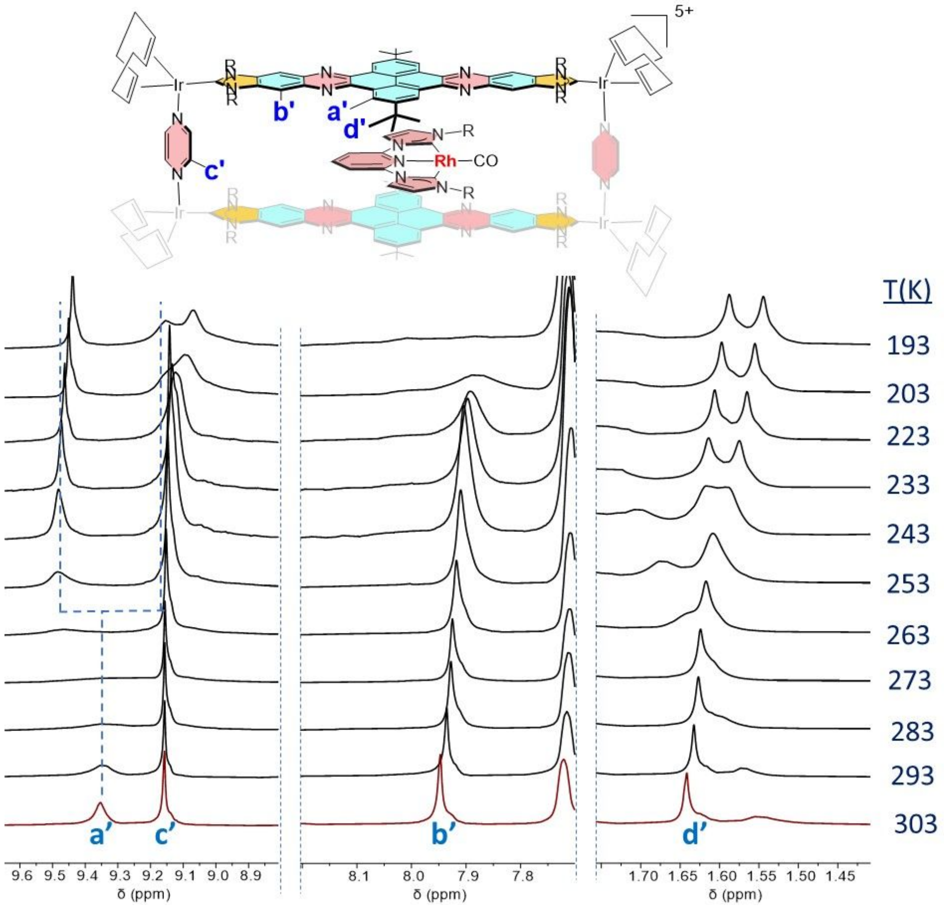

Figure S12. Three selected regions of the VT-^{1}H NMR studies (500 MHz) of complex **2@1** in CD_2Cl_2 with temperatures ranging from 193-303 K.

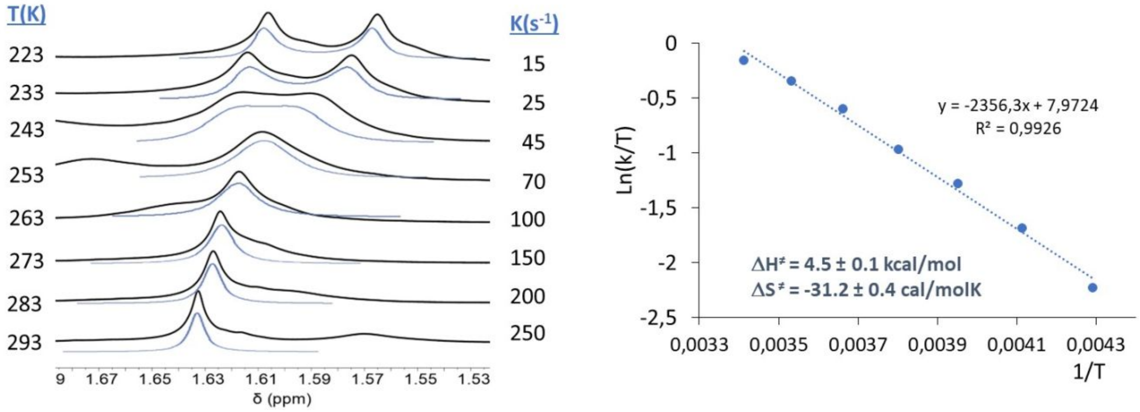

Figure S13. Study of the dynamic behavior of complex **2@1** by variable temperature ^{1}H NMR spectroscopy. All spectra were recorded in CD_2Cl_2. The calculation of the exchange rates was performed by lineshape analysis using SpinWorks 4.0. Simulated spectra (in blue) are shown below the experimental ones (in black). Eyring plot is shown on the right, together with the non-linear fitting analysis and the resulting activation parameters.

6. DOSY experiments

The experiments were carried out on CD_2Cl_2, at constant concentrations of 5 mM on a Varian Innova 500 MHz.

Complexes	G (m^2/s)
**1^2**	$5.58\ 10^{-10}$
**2**	$1.07\ 10^{-9}$
**2@1**	$5.31\ 10^{-10}$

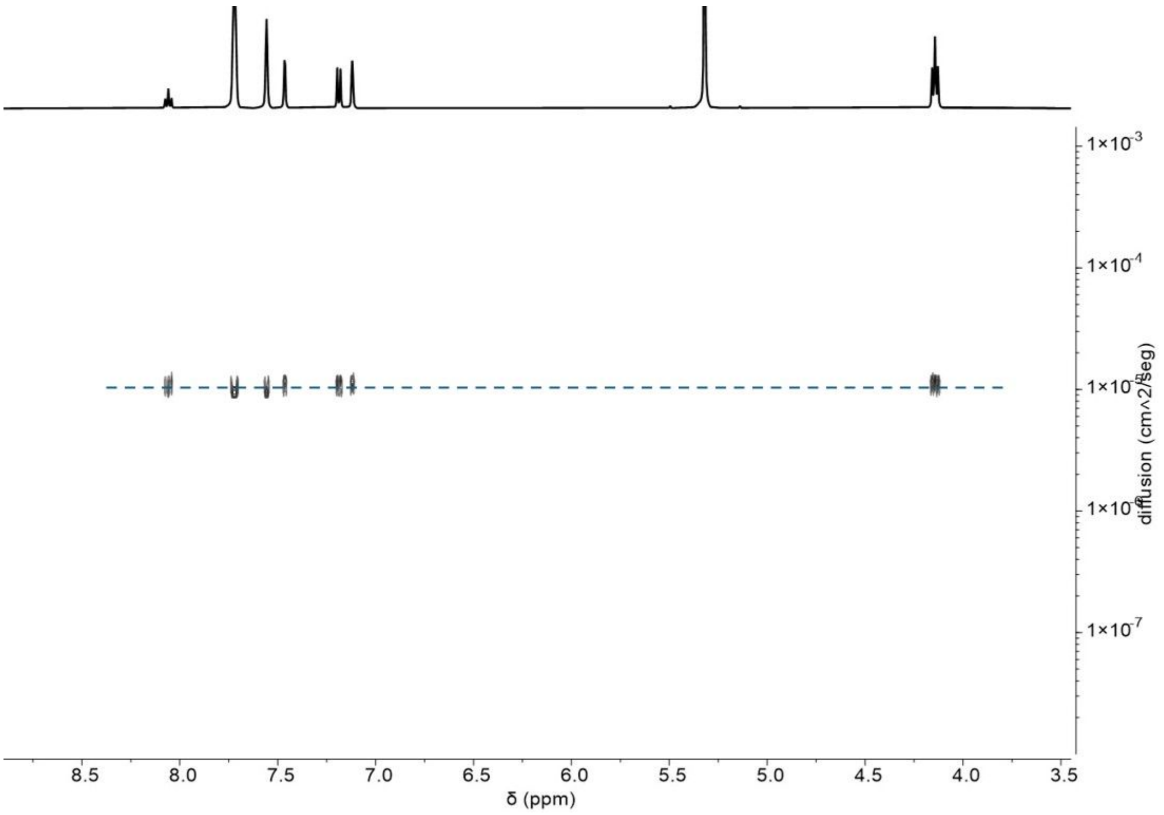

Figure S14. DOSY NMR spectrum (500 MH_Z, 5 mM) of **2** in CD_2Cl_2 at 298 K.

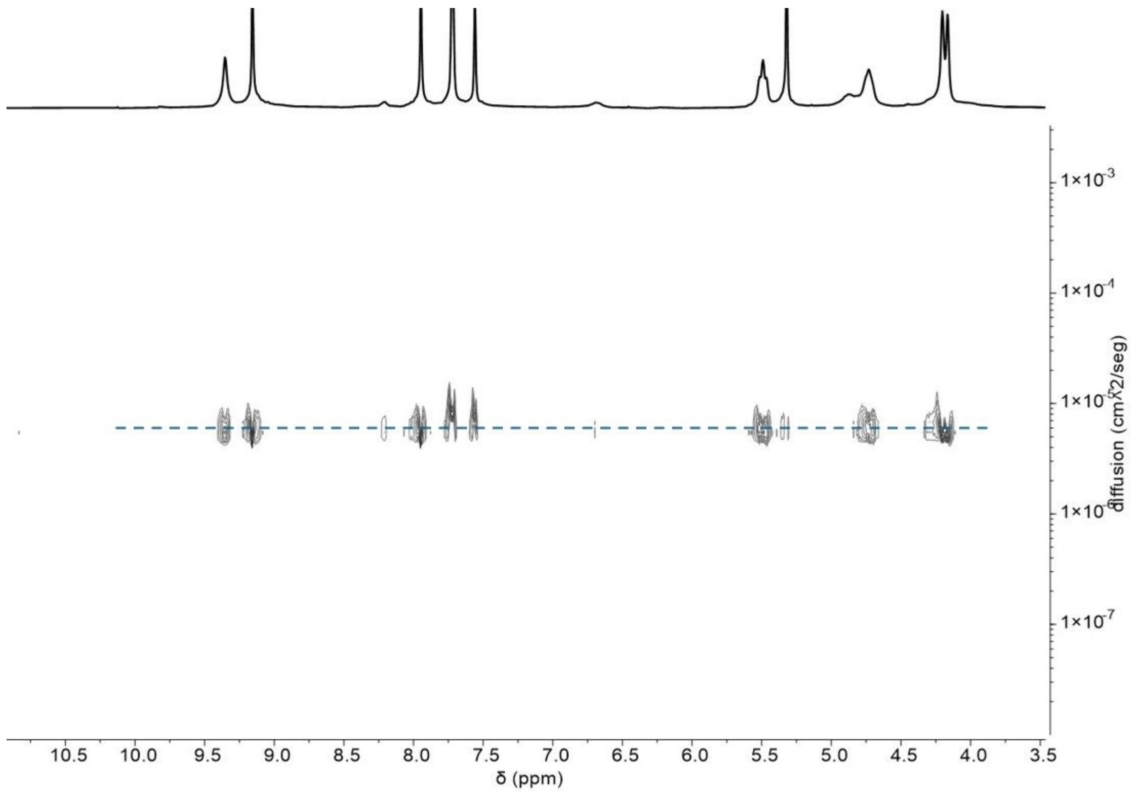

Figure S15. DOSY NMR spectrum (500 MHz, 5 mM) of **2@1** in CD_2Cl_2 at 298 K.

7. Kinetic studies: oxidative addition of MeI

NMR tubes were charged with CD_2Cl_2 (0.5 mL) containing one of three sample compositions: (i) complex **2** (2 mM), (ii) a 2:1 molar mixture of **2** and **1** (2 mM and 1 mM, respectively), or (iii) a 1:1 molar mixture of **2** and **1** (2 mM each). To each tube was added a solution of CH_3I in CD_2Cl_2 (60 µL, 0.76 M, 48 equiv.), and the resulting mixture was immediately placed in the spectrometer. ^{1}H NMR spectra were collected at 313 K using an arrayed experiment with a data point recorded every 5 min (8 scans per data point, relaxation delay = 3 s). The progress of the oxidative addition reaction was monitored by integration of the Rh–CH_3 resonance as a function of time. Whereas oxidative addition proceeded in samples (i) and (ii), no formation of the oxidative addition product was observed in sample (iii), indicating that a 1:1 ratio of **2** to **1** completely suppresses the reaction.

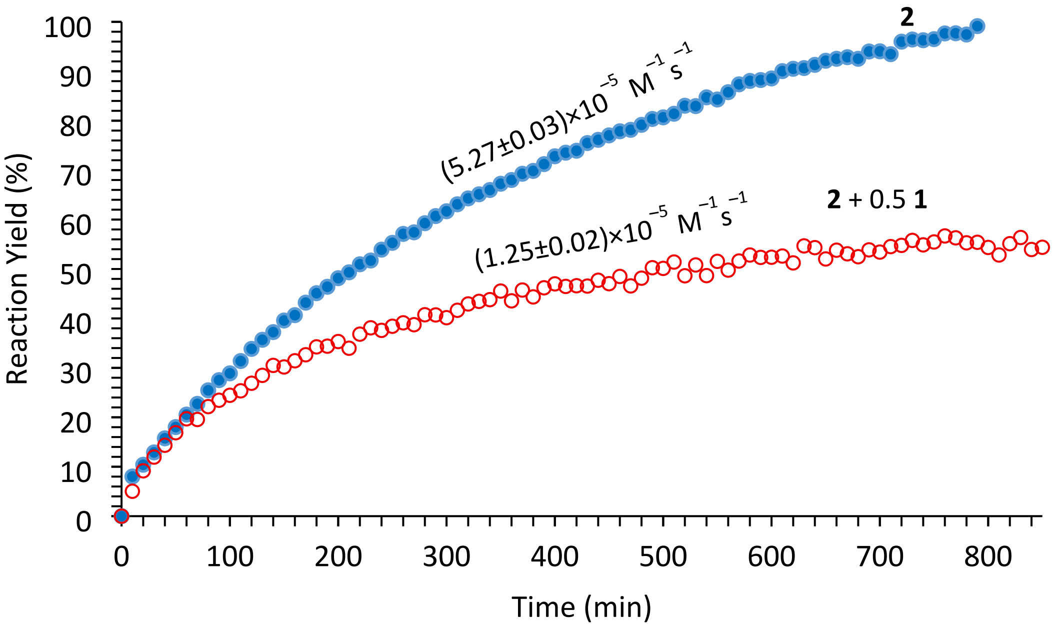

Figure S16. Time-dependent reaction profiles of the reaction of **2** and a 2:1 molar mixture of **2** and **1** with CH_3I at 313 K. The pseudo-first order kinetic constant is also depicted in the graphic. For the reaction performed adding one equivalent of **1**, no formation of Rh(III) complex was observed (not shown in the graphic).

<u>Control Experiment: Stability of **1** toward CH_3I</u>

To confirm that **1** does not react with CH_3I under the reaction conditions, a control experiment was performed. An NMR tube was charged with a solution of **1** (2 mM) in CD_2Cl_2 (0.5 mL), and CH_3I in CD_2Cl_2 (60 µL, 0.76 M, 48 equiv.) was added. The ^1H NMR spectrum of the mixture recorded after 24 h showed no changes relative to the spectrum of **1**, confirming that **1** is unreactive toward CH_3I under these conditions.

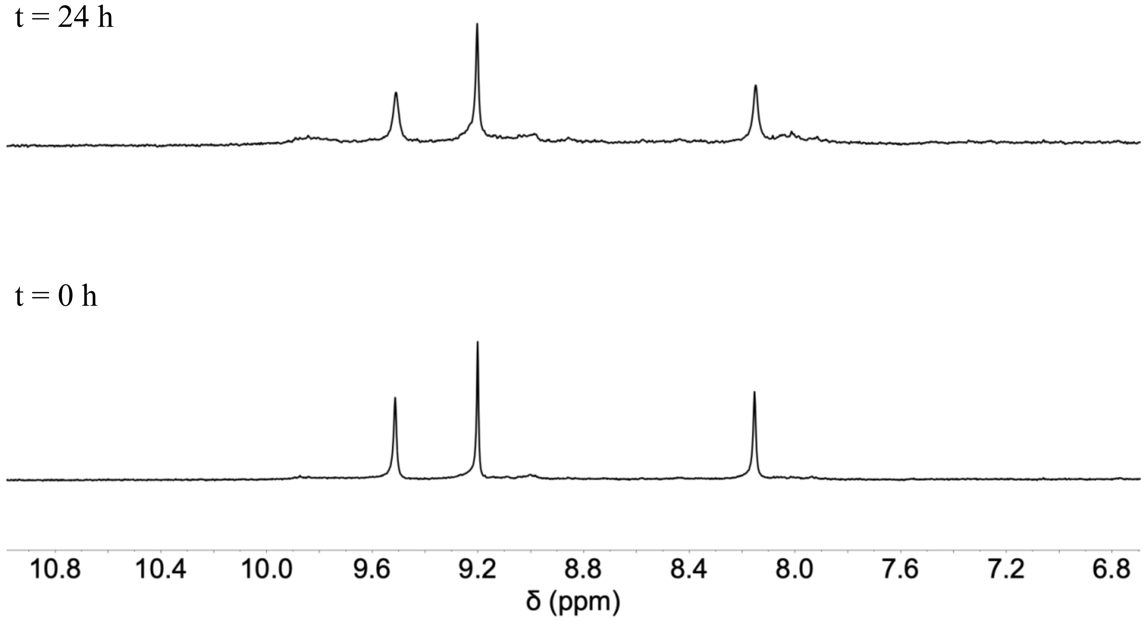

Figure S17. ¹H NMR spectra (400 MHz) of **1** in the presence of CH_3I (48 equiv.) at t = 0 h and t = 24 h, confirming the integrity of **1** under the reaction conditions.

8. References

1. Thordarson, P., Determining association constants from titration experiments in supramolecular chemistry. *Chem. Soc. Rev.* **2011,** *40*, 1305-1323.

2. Ibáñez, S.; Salvá, P.; Dawe, L. N.; Peris, E., Guest-Shuttling in a Nanosized Metallobox. *Angew. Chem., Int. Ed.* **2024**, e202318829
